# O011. Patients with “prolonged aura” do not show clinical or demographic differences from the patients with “typical aura”

**DOI:** 10.1186/1129-2377-16-S1-A67

**Published:** 2015-09-28

**Authors:** Michele Viana, Mattias Linde, Grazia Sances, Natascia Ghiotto, Elena Guaschino, Marta Allena, Salvatore Terrazzino, Giuseppe Nappi, Peter J Goadsby, Cristina Tassorelli

**Affiliations:** Headache Science Centre, C. Mondino National Neurological Institute, Pavia, Italy; Department of Neuroscience, Norwegian University of Science and Technology, Trondheim, Norway; Norwegian Advisory Unit on Headaches, St. Olavs University Hospital, Trondheim, Norway; Università del Piemonte Orientale “A. Avogadro”, Department of Pharmaceutical Sciences, Novara, Italy; Headache Group, NIHR-Wellcome Trust Clinical Research Facility, King's College, London, UK; Department of Brain and Behavioural Sciences, University of Pavia, Pavia, Italy

## Background

A recent systematic review of the duration of migraine aura reported that aura symptoms may last longer than one hour in a significant proportion of patients[1]. Here we investigated in a prospective diary-aided study whether patients with a “prolonged aura” (PA - an aura in which there is at least one symptom lasting for more than one hour) are different from the patients with a “typical aura” (TA).

## Methods

We recruited 176 consecutive patients affected by migraine with aura at the Headache Centres of Pavia and Trondheim. The study received approval by the local Ethics Committees. All patients signed an informed consent form. Fifty-four patients completed the study recording prospectively the characteristics of three consecutive attacks in an *ad hoc* aura diary that included the time of onset and the end of each aura symptom and the headache. We also collected demographic and clinical variables of each patient including age, gender, presence of headache associated with aura, frequency of migraine with aura attacks, age at onset of migraine with aura, duration of illness, co-occurrence of migraine without aura or tension-type headache, age of migraine without aura onset, use of a migraine preventive therapy, family history for migraine with aura and white matter lesions at MRI in the analysis. We performed an analysis to evaluate if there was any demographic or clinical variable associated with having suffered from at least one PA out of three attacks.

## Results

Fifty-four patients completed the study recording in a diary the characteristics of three consecutive auras (n=162 auras). Fourteen out of 54 patients (26%) had at least one PA, while 30 patients (74%) had three TA. In univariate analyses, none of the clinical or demographic parameters was significantly associated with the fact of having experienced a PA (Table 1).

**Table 1:** Association between clinical variables and the condition of having suffered of at least one migraine with prolonged aura out of three attacks: univariate analysis.

Clinical variable	All patients (n=54)	Patients without prolonged aura	Patients with at least one prolonged aura	P value
**Sex**				
Female Male	45 (83.3) 9 (16.7)	32 (80.0) 8 (20.0)	13 (92.9) 1 (7.1)	0.487
**Age, years (SD)**	39.6 (14.5)	41.2 (14.4)	34.8 (14.0)	0.153
**Age at MwA onset, years (SD), n=52**	23.4 (11.5)	24.2 (12.2)	21.3 (9.1)	0.571
**Frequency of MwA, attacks/year (SD)**	23.9 (27.6)	24.1 (29.6)	23.3 (22.0)	0.866
**Duration of MwA, years (SD), n=52**	15.6 (12.7)	17.1 (12.6)	11.3 (12.3)	0.079
**Aura with headache**				
on 3/3 attacks on 0/3 attacks on 1/3 or 2/3 attacks	46 (85.2) 3 (5.6) 5 (9.3)	32 (80.0) 3 (7.5) 5 (12.5)	14 (100) 0 (0) 0 (0)	0.193
**Co-occurrence of MwoA n=53**				
No Yes	15 (28.3) 38 (71.7)	10 (25.6) 29 (74.4)	5 (35.7) 9 (64.3)	0.710
**Age at MwoA onset, years (SD), n=38**	17.8 (8.6)	18.3 (8.7)	16.2 (8.6)	0.327
**Frequency of MwoA attacks/month (SD), n=38**	5.2 (5.3)	5.4 (5.7)	4.6 (1.4)	0.904
**Co-occurrence of tension type headache**				
No Yes	46 (85.2) 8 (14.8)	36 (90.0) 4 (10.0)	10 (71.4) 4 (28.6)	0.213
**Familiarity for aura, n=52**				
No Yes	40 (76.9) 12 (23.1)	28 (73.7) 10 (26.3)	12 (85.7) 2 (14.3)	0.588
**Preventive prophylaxis, n=53**				
No Yes	33 (62.3) 20 (37.7)	26 (66.7) 13 (33.3)	7 (50.0) 7 (50.0)	0.434
**White Matter Changes at MRI, n=45**				
No Yes	34 (75.6) 11 (24.4)	23 (71.9) 9 (28.1)	11 (84.6) 2 (15.4)	0.604

Prolonged aura: aura with at least one symptom lasting for more than 60 minutes.

MwA: migraine with aura; MwoA: migraine without aura.

## Conclusions

For the first time we demonstrate that patients with “prolonged aura” have no demographic and clinical differences with patients with “typical aura”. These data support the need to review the ICHD criteria for migraine with aura.

Written informed consent to publication was obtained from the patient(s).

## Conflict of interest

None.
